# London Hospital Nurses

**Published:** 1918-09-21

**Authors:** W. A. Chapple

**Affiliations:** House of Commons


### THE EDITOR'S LETTER-BOX.

**[Correspondence on all subjects is invited, but we cannot in any way be responsible for the opinions expressed by our correspondents, who must give their name and address as a guarantee of good faith, but not necessarily for publication. Correspondents are reminded that brevity of style and conciseness of statement greatly facilitate early insertion.]**

### London Hospital Nurses.

*To the Editor of* The Hospital.

Sir,—You have given considerable space to the treatment of its nurses by the London Hospital. You will do a great service in the direction of an urgent reform if you will keep the subject clear and free from all side issues. These facts are admitted by the London Hospital authorities : (1) That probationers sign on for four years, two years of training and two years of "service." (2) That at the end of two years they get a certificate of two years' training. (This is of no service to the nurse; it cannot be used by her, and only serves to enable the London Hospital to advertise in the *Times* "trained nurses can be had immediately.") (3) That these nurses are sent out after the end of their second year's training to attend private cases, receiving during their third year 13s. 5d. per week, while they earn £2 12s. 6d. for the hospital. (4) That the hospital has profited to the extent of £5,000 per annum by the "service" of the private staff. All this is admitted without a blush by those responsible. No other great hospital in the kingdom is guilty of this exploitation of defenceless and sweated women.

If these young women are "trained nurses" 13s. 5d. per week is a sweated wage; if they are not "trained nurses" £2 12s. 6d. per week is an exorbitant charge to the sick.

It is a cruel imposition on eager, conscientious young women anxious to get all the training and experience that so great and good a hospital as the London can provide, to take them from their training in the wards and farm them out for profit—appropriating not less, often more, than 29s. per week of their earnings. 'Bus girls would not submit to this : nurses must. Where is chivalry? I promise you and the London Hospital that, now that women have the vote, no candidate at the next election shall be ignorant of these facts.—Yours faithfully,

W. A. Chapple.

House of Commons, September 10, 1918.

